# *Salmonella enterica* serovar Schwarzengrund: Distribution, Virulence, and Antimicrobial Resistance

**DOI:** 10.3390/microorganisms13010092

**Published:** 2025-01-06

**Authors:** Monique A. Felix, Jing Han, Bijay K. Khajanchi, Yasser M. Sanad, Shaohua Zhao, Steven L. Foley

**Affiliations:** 1 Division of Microbiology, National Center for Toxicological Research, U.S. Food and Drug Administration, Jefferson, AR 72079, USA; moniquefelix148@gmail.com (M.A.F.); jing.han1@fda.hhs.gov (J.H.); bijay.khajanchi@fda.hhs.gov (B.K.K.); sanady@uapb.edu (Y.M.S.); 2 Department of Agriculture, University of Arkansas at Pine Bluff, Pine Bluff, AR 71601, USA; 3 Office of Applied Research and Safety Assessment, Center for Food Safety and Applied Nutrition, U.S. Food and Drug Administration, Laurel, MD 20708, USA; 4 Office of Applied Research, Center for Veterinary Medicine, U.S. Food and Drug Administration, Laurel, MD 20708, USA; shaohua.zhao@fda.hhs.gov

**Keywords:** IncFIB-IncFIC(FII) plasmids, genotypic analyses, virulence database, plasmid transfer genes, phylogenetics

## Abstract

The global incidence of *Salmonella enterica* serovar Schwarzengrund has risen in recent years. This serotype has been isolated from poultry, retail meat, and other food products, leading to multiple outbreaks. Alongside the increase in infections, there are growing concerns about the increasing levels of antimicrobial resistance (AMR) among *S.* Schwarzengrund strains. This study aims to better understand the genetic factors possibly contributing to the rising prevalence of *S.* Schwarzengrund by analyzing the sequences of 2058 isolates from both human patients (N = 313) and food- and animal-associated sources, including chicken (N = 1145), turkey (N = 300), pork (N = 132), and other sources (N = 168). Data were obtained from GenBank and analyzed for AMR genes using AMRFinder. Additionally, putative virulence genes and plasmid transfer genes were assessed using the Virulence and AMR Plasmid Transfer Factor Database. AMR genes were found in 1269 (61.7%) of the isolates, with a total of 2478 AMR genes among the isolates, the most common being *aph(3″)-Ib* (N = 969, 47.1%), *tet(A*) (N = 190, 9.2%), and *sul2* (N = 150, 7.3%), which are responsible for resistance to aminoglycoside, tetracycline, and sulfonamide, respectively. Additionally, 1060 (51.5%) isolates carried multiple plasmid transfer genes associated with IncFIB-FIC(FII) plasmids. Other plasmid types found in at least 1% of the strains included IncI1 (N = 101, 4.9%), IncHI2 (N = 62, 3.0%), or IncHI1 (N = 24, 1.2%). The virulence gene profiles of human isolates showed diversity but largely overlapped with those from different food sources. Notably, the aerobactin iron acquisition genes, associated with *Salmonella*’s virulence and colonization, were highly prevalent among chicken isolates (N = 1019, 89.0%) but less frequent in isolates from other sources (N = 65, 7.2%). IncFIB-FIC(FII) plasmids, commonly harboring the aerobactin operon, were highly prevalent among chicken-related isolates and present in about 10% of human isolates. The diverse plasmid, AMR, and virulence gene profiles in human-associated isolates suggest that multiple factors may contribute to the increased virulence in *S.* Schwarzengrund.

## 1. Introduction

In the United States (U.S.), there are over one million cases of *Salmonella* infection annually, leading to approximately 20,000 hospitalizations and nearly 400 deaths, according to estimates by the Centers for Disease Control and Prevention (CDC) [1]. *Salmonella* infects and invades the intestinal tract and is disseminated through the excretion of feces. The disease spectrum of salmonellosis in humans ranges from relatively mild food poisoning and gastroenteritis to more severe illnesses that can include enteric fever and septicemia [2]. Salmonellosis is often associated with fecal contamination of water or food sources such as meat, poultry, and eggs, as well as through the contact with cross-contaminated surfaces and utensils in poor hygienic conditions. Common signs and symptoms of salmonellosis include stomach cramps, bloody stools, chills, diarrhea, fever, headaches, muscle pain, nausea, vomiting, and dizziness [3,4]. *Salmonella* infections are categorized as either typhoidal (human-specific) or non-typhoidal (which affect a broad range of hosts) [2,5]. In the U.S., most infections are caused by non-typhoidal *Salmonella,* which infects both humans and animals. One serovar that causes non-typhoidal infections is *S*. Schwarzengrund [6], the primary focus of this research.

*S.* Schwarzengrund has been an emerging serovar over the last few decades, rising to be one of the more common serovars in countries such as Thailand, Slovakia, New Zealand, and Venezuela [6]. The first report of *S.* Schwarzengrund in the U.S. was in the early 1950s. In this case, nine-day old turkeys from a farm in Indiana were sent to a diagnostic lab. At the farm, 754 of 13,000 turkeys (5.8%) died in the first 4 days following infection. *S.* Schwarzengrund strains were subsequently isolated from the liver of the turkey. This isolation was the first in the U.S. and the second recorded worldwide [7]. Since that time, the number of *S*. Schwarzengrund infections in food animals and humans has increased. Data from the CDC showed that the isolation rates of *S*. Schwarzengrund had increased over the years, especially in the upper eastern region of the U.S. [8]. From 1968 to 2011, 77.7% of reported human isolates, as documented by the CDC, originated from stool samples; however, 9.8% were from more invasive sources such as blood, cerebral spinal fluids, joints, or wounds/abscesses, and 3.6% were from urine [8]. These high rates of extraintestinal infections are particularly alarming given that they often require antimicrobial therapy, making AMR in this serovar especially concerning.

Globally, the prevalence of *S*. Schwarzengrund has also increased. In Asian countries, including Japan and Thailand, its prevalence in chickens increased from the mid-1990s to the mid-2000s, and it has maintained its status as a top serovar from chickens in Japan [9,10,11,12]. *S*. Schwarzengrund has also been among the top four most prevalent serovars isolated from human patients in Japan in recent years [13]. Pulsed-field gel electrophoresis (PFGE) analyses of *S*. Schwarzengrund isolates revealed that antibiotic-resistant strains were spreading among broiler chickens and retail chicken meat with cross contamination occurring during processing, likely through contaminated transport crates and utensils [12]. In the U.S., *S*. Schwarzengrund ranks among the top five *Salmonella* serovars isolated from pig farms and is one of the top fifteen serovars found in food, animals, and humans in Thailand, Slovakia, New Zealand, and Venezuela [6].

A study conducted by Chen et al. (2010) found that *S*. Schwarzengrund were more prevalent during the summer compared to winter and on certain parts of the chicken, such as the buttock and neck [14]. A study by Duc et al. (2020) showed that 17.8% (546/3069) of broiler chicken cecal samples collected between 2003 and 2016 were positive for *Salmonella* spp., and 21.3% (116/546) were identified as *S*. Schwarzengrund, a significant increase compared to 2.1% (5/243) in a previous study conducted from 2009 to 2012 [15]. In the U.S., *S*. Schwarzengrund outbreaks among humans have been linked to contact with dry pet food and consumption of contaminated ground turkey [16,17].

AMR in *S*. Schwarzengrund is of particular concern, as it complicates the treatment of invasive infections. Internationally, there has been a rise in multidrug-resistant (MDR) *S.* Schwarzengrund strains which can resist antibiotics such as tetracyclines, sulfonamides, aminoglycosides, β-lactams, and fluoroquinolones [18,19,20]. In Brazil, 11% of isolates from poultry carried the extended-spectrum β-lactamase (ESBL) resistance gene *bla*_CTX-M-2_, which encodes resistance to critical antimicrobials [21]. In Taiwan, 30% of S. Schwarzengrund strains from retail chicken meat were resistant to antibiotics, with 15.8% resistant to quinolones and 28.1% resistant to ciprofloxacin [14]. Notably, *S.* Schwarzengrund was the first *Salmonella* serotype in the U.S. found to be resistant to fluoroquinolones, following a case involving a patient who had traveled to the Philippines [21].

The pathogenicity of *S.* Schwarzengrund is driven by a wide range of virulence factors, including type 3 secretion systems (T3SSs), encoded by *Salmonella* Pathogenicity Islands (SPIs) 1 and 2, which facilitate invasion and survival within host cells [2]. Additional virulence genes are found on both chromosomally and plasmid-encoded gene clusters, further enhancing the bacterium’s ability to infect hosts. Plasmids also play a critical role in *S.* Schwarzengrund’s AMR and virulence. Fusion plasmids, such as IncFIB-IncFIC(FII), carry genes for iron acquisition (*iucABCD* and *iutA* of the aerobactin operon), virulence factors, and AMR. These plasmids can enhance the bacterium’s survival under nutrient-limited conditions and facilitate horizontal gene transfer, spreading AMR traits across bacterial populations [22]. In addition to their fusion plasmids, distinct IncFII and IncFIC plasmids are also found in *S.* Schwarzengrund; these can facilitate horizontal transfer of various AMR genes such as the extended-spectrum β-lactamase genes [22,23,24,25]. In addition to these IncF-related plasmids, other AMR-associated plasmid types such as IncI1, IncHI1, and IncHI2 plasmids have also been detected among *S.* Schwarzengrund isolates.

Given their rising prevalence, and the AMR and virulence profiles of many *S*. Schwarzengrund strains, especially in food animal hosts, there is a need for more research to better understand their genetic diversity. The goal of this project was to provide deeper insights into the virulence factors, AMR genes, and plasmids present in *S.* Schwarzengrund by conducting an extensive analysis of over 2000 publicly available whole genome sequences from the GenBank database.

## 2. Materials and Methods

### 2.1. Bacterial Strains Analyzed

To investigate the genetic characteristics of *S.* Schwarzengrund, sequenced isolates were identified using the National Center for Biotechnology Information (NCBI) Pathogen Detection Isolate Browser. Relevant metadata, including isolate source/type, geographical location, year of isolation, and SNP cluster information, were extracted from both GenBank and the NCBI Pathogen Detection Isolate Browser (https://www.ncbi.nlm.nih.gov/pathogens/ (accessed on 21 May 2024)). The whole genome sequence (WGS) FASTA files of 2058 *S*. Schwarzengrund isolates were extracted from GenBank in November of 2022. These isolates included samples from human patients (N = 313) and food- and animal-associated sources, including chicken (N = 1145), turkey (N = 300), and pork (N = 132), representing all the available *S*. Schwarzengrund WGSs at the time of data collection.

### 2.2. Virulence, Plasmid, and AMR Gene Detection

Putative virulence genes and plasmid transfer genes were identified using the Virulence and AMR Plasmid Transfer Factor Database (https://virulence.fda.gov) [26,27]. The WGS FASTA files for each strain were uploaded into the database and analyzed using the *Salmonella* Virulence Factor Comparison and Plasmid Transfer Gene Comparison tools, respectively. For AMR gene detection, data were either extracted directly from the NCBI Pathogen Detection Isolate Browser or, where unavailable, the WGS FASTA files were analyzed with AMRFinder (ver. 26.1) within the GalaxyTrakr platform [28,29]. This allowed for the identification of AMR genes across all of the *S.* Schwarzengrund isolates.

### 2.3. Data Analysis

The sample metadata, along with presence/absence data for virulence, and AMR and plasmid transfer genes were extracted from the respective analysis tools and formatted as delimited files. These were then imported into Excel (Microsoft, Redlands, WA, USA), for initial descriptive statistics of prevalence of AMR, putative virulence, and plasmid transfer-associated genes. For more detailed analyses, the data were uploaded into BioNumerics (ver. 8.1; Applied Maths, Austin, TX, USA). BioNumerics was used for phylogenetic analyses based on the presence/absence of genes across different metadata categories. These analyses included the identification of polymorphic characters (i.e., those genes detected in 1–99% of isolates), performing principal components analysis (PCA), and constructing minimal spanning trees using default parameters for the character data sets.

## 3. Results and Discussion

### 3.1. S. Schwarzengrund Demographics

**Figure 1:**
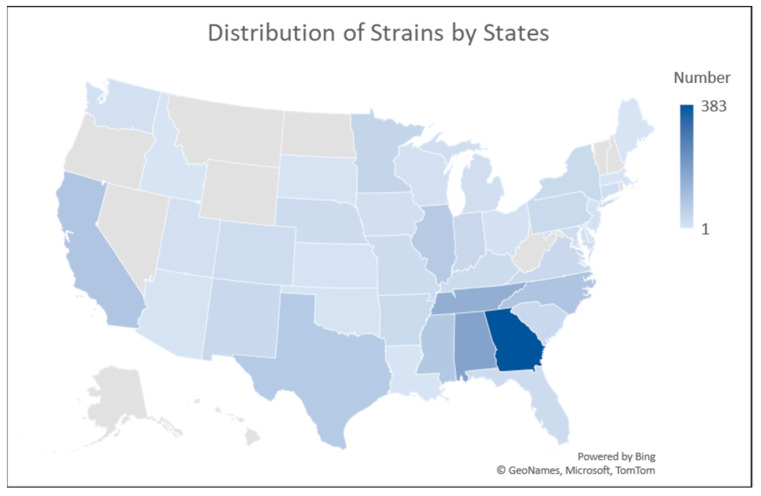
Distribution of *S.* Schwarzengrund strains isolated within the United States. The heatmap to the right of the figure indicates the numbers of isolates from each state that were present in the GenBank dataset.

*S.* Schwarzengrund infections have been increasing globally over the last several years; however, most strains that have undergone whole genome sequencing are from the United States [6,15]. In this dataset, the vast majority (85.6%, N = 1762) of the strains originated in the U.S., followed by the United Kingdom (8.5%, N = 174) (Table 1). The majority of the U.S. isolates were collected through surveillance efforts by the Food and Drug Administration (FDA) and the U.S. Department of Agriculture’s Food Safety and Inspection Service (FSIS). When looking at the isolation locations within the U.S., the southeastern states, particularly Georgia (GA; 21.7% of U.S. strains, N = 383), Alabama (AL; 9.9%, N = 175), Tennessee (TN; 8.2%, N = 144), and North Carolina (NC; 4.9%, N = 87), reported the highest number of isolates (Figure 1). In terms of isolate sources, chicken-related samples dominated (55.6%, N = 1145), followed by human patients (15.2%, N = 313), turkey (14.6%, N = 300), and porcine-related samples (6.4%, N = 132). Among the U.S. isolates, food animal sources were even more prominent, with chicken accounting for 62.9% (N = 1102), turkey, 17.0% (N = 299), and porcine, 7.0% (N = 124). Conversely, the percentage of human isolates from the U.S. was lower (6.0%, N = 106) than the overall percentage. Interestingly, the largest proportion of isolates from human patients was from the U.K., at 52.7% (N = 165). In terms of state-level comparisons within the U.S., isolates from GA, AL, and TN were predominantly chicken-related, while NC isolates were more often turkey- and pork-related. Unfortunately, the absence of detailed state-level data for many human isolates, likely due to privacy concerns, limits further comparisons. This lack of metadata complicates attempts to correlate human isolates with food source origins, emphasizing the need for better documentation of human cases to improve understanding of transmission pathways.

### 3.2. Antimicrobial Resistance

**Figure 2:**
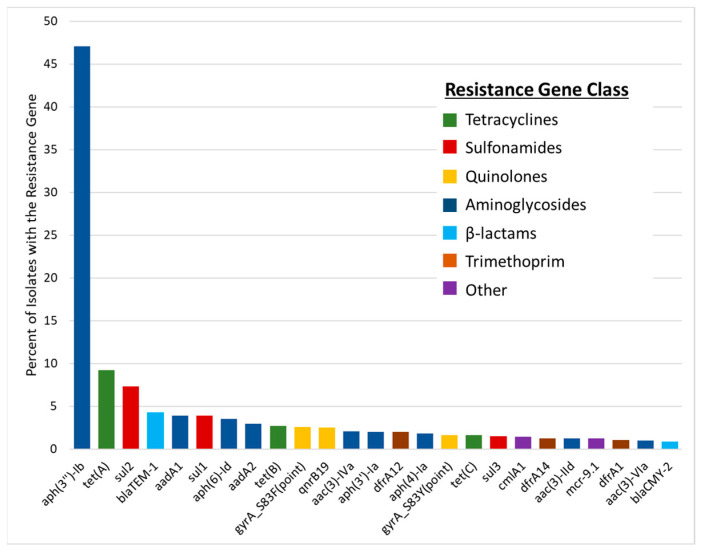
Percent of *S.* Schwarzengrund isolated in this study that carried the respective AMR genes. The bars are color-coded based on antimicrobial classes where resistance genes were detected.

**Figure 3:**
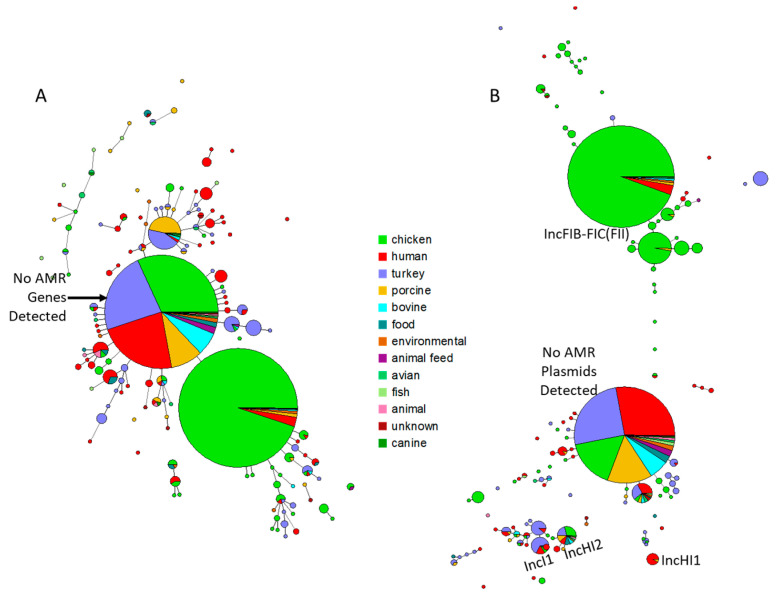
Minimum spanning tree analyses based on the AMR (panel (**A**)) and plasmid transfer gene (**B**) profiles of the isolates included in this study. The trees are color-coded based on isolation source. The relative size of the circles is proportional to the group size. In panel (**A**), the larger circle in the middle represents the strains without an identified AMR gene. In panel (**B**), the larger ball near the bottom is the group without a detected AMR plasmid. The groups representing the major plasmid types are annotated. The colors are described by the legend included in the figure.

AMR genes were relatively common among the isolates, with 1269 (61.7%) carrying at least one AMR gene. Overall, there were 2478 AMR genes among the isolates, the most commonly observed being *aph(3″)-Ib* (aminoglycoside; N = 969, 47.1%), followed by *tet(A)* (tetracycline; N = 190, 9.2%) and *sul2* (sulfonamide; N = 150, 7.3%) (Figure 2). Other notable AMR genes included *bla*_TEM-1_ (β-lactamase), *sul1* (sulfonamide), and aminoglycoside resistance genes *aadA1* and *aph(6)-Id,* each present in at least 3% of the strains. Detailed results of the AMR profiles are presented in Table S2. There were two major AMR gene profiles: those without any detectable AMR genes (upper ball in Figure 3A and Figure S1A) and those carrying only *aph(3”)-Ib*. Strains carrying *aph(3”)-Ib* were predominantly from chickens and frequently associated with IncFIB-FIC(FII) plasmids, which is consistent with our earlier findings [29]. This plasmid has been shown to be transmissible in vitro, raising concerns about the potential spread of resistance within poultry populations and, subsequently, to humans.

Although the majority of isolates fell into two main AMR profiles, smaller profiles exhibited more diverse AMR gene combinations, particularly among non-chicken sources. The high prevalence of *aph(3”)-Ib* among chicken-related strains, and its association with transmissible plasmids, highlights the potential risk of widespread of antimicrobial resistance within the poultry industry and its broader impact on human health, especially in the context of treatment efficacy for related infections [30].

When compared to AMR profiles in other *Salmonella* serovars, the predominance of *aph(3”)-Ib* in *S.* Schwarzengrund isolates highlights a distinctive pattern of resistance, suggesting that plasmid-mediated transmission may play a more significant role in this serovar’s resistance profile. Although *aph(3”)-Ib* dominates the AMR profiles, the presence of β-lactamase (*bla*_TEM-1_) and sulfonamide (*sul1*) resistance genes in over 3% of isolates further underscores the need for continued surveillance, particularly for emerging multidrug-resistant strains in non-poultry sources.

### 3.3. Plasmid Characterization

Among the identified plasmid types, approximately 51.5% (N = 1060) carried multiple plasmid transfer genes associated with IncFIB-FIC(FII) plasmids, while other plasmid types, such as IncI1 (N = 101, 4.9%), IncHI2 (N = 62, 3.0%), or IncHI1 (N = 24, 1.2%), were detected in at least 1% of the strains (Figure 3B and Figure S1B). The high prevalence of IncFIB-FIC(FII) plasmids is of particular concern given their carriage of a range of virulence and antimicrobial resistance genes, potentially contributing to increased pathogenicity and resistance spread. The detailed results of the plasmid transfer gene analysis are presented in Table S3 in the supplemental data. The approach of using the transfer genes associated with the different plasmid types has value in both predicting the plasmid types present and identifying potential limitations on conjugal ability if genes are absent. The current study did not evaluate conjugation ability; however, our recent study has demonstrated that some of the IncFIB-FIC(FII) fusion plasmids from *S.* Schwarzengrund can transfer under in vitro conditions, although not in all cases [30], highlighting the risk of horizontal gene transfer in the agriculture environment [31]. The presence of transmissible plasmids in poultry isolates may have important implications for both food safety and public health [32]. Given the predominance of these plasmids in isolates from chicken-related sources, further investigation into their mobility and contribution to the spread of AMR genes across different environments is warranted.

### 3.4. Virulence Genetics Characterization

The virulence gene profiles were determined using the *Salmonella* Virulence Factor Comparison tool. Of the 590 genes examined, 43.9% (N = 259) were present in all isolates (Table S4). These genes included those associated with SPIs 1–5 and 9, as well as certain fimbrial genes (e.g., *csg* and *std* operons). Additionally, 23.1% (N = 136) were present in more than 99% of the strains. In contrast, 15.6% (N = 92) of the genes were detected in less than 1% of the strains, including 44 genes that were absent in all strains. These low prevalence and absent genes include those associated with different SPIs (e.g., SPI-7 associated with *S.* Typhi) and many of the *Salmonella* genomic island (SGI)-1 genes. The remaining 17.5% (N = 103) of genes, which represented those present in between 1 and 99% of the isolates, were considered polymorphic characters which provided useful markers for comparing the virulence gene profiles across different isolates from different sources. As shown in Figure S2, phylogenetic analyses of virulence profiles revealed that most chicken-related isolates clustered into four large groups, each containing over 70 strains. In addition, there were two large groups that contained over 300 strains each from multiple different sources, including many isolates from human patients. These results highlight that the human isolates exhibited greater diversity of virulence gene profiles yet frequently overlapped with those from different food/animal sources. One of the key differences between the four predominantly chicken clusters and those with the more diverse sources is that isolates from chickens carried a higher prevalence of IncFIB-FIC(FII) plasmid-associated virulence factors, such as the aerobactin iron acquisition genes (*iucABCD* and *iutA*) and *traT*. These genes were significantly more common in chicken-related isolates (88.5%, N = 1019) than in those from other sources (7.1%, N = 65) (Table 2). Certain genes, such as *allD, envF, sseK1*, and *sspH2*, as well as several fimbrial operons (including *stc, ste, stf,* and *stk*), were more commonly found in strains from human patients compared to those from chicken sources. These fimbrial genes were particularly interesting because strains exhibited different combinations of operons (*sta, stb, stc, ste, stf, stk,* and *stj*), while *std, sth,* and *sti* were present in nearly all (>99%) strains, and the *stg* operon was absent in all strains. When looking at the VF profiles based on geographic location, the vast majority of the strains carrying the IncFIB-FIC(FII) plasmids and associated VFs originated from the U.S., while those lacking the plasmids were from a wider range of locations.

**Figure 4:**
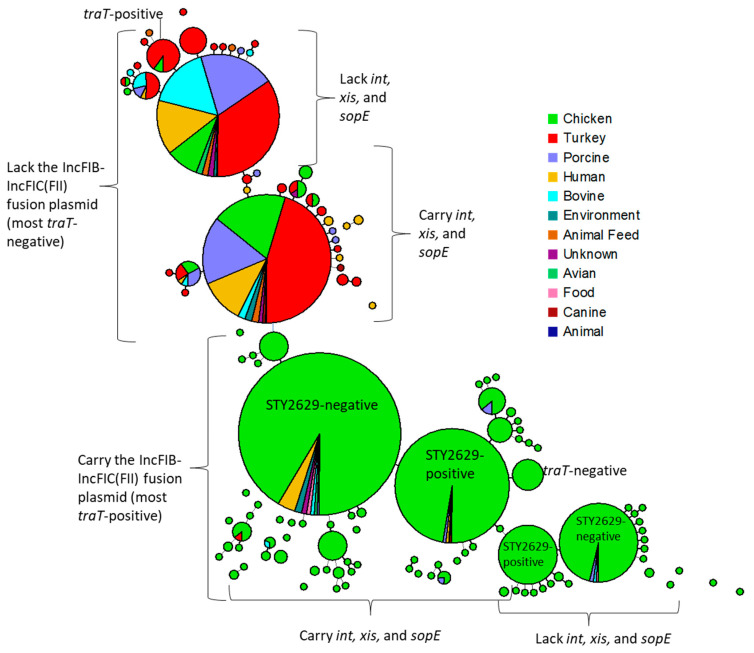
Minimum spanning tree analyses of strains originating within the United States based on their VF profiles. The trees are color-coded based on isolation source and the relative size of the circles is proportional to the group size. Key VF traits of the different clusters are annotated within the figure, including the presence or absence of key genetic factors. The colors are described by the legend included in the figure.

As nearly 86% of the isolates originated from the U.S., we conducted a more detailed comparison of the VF profiles of these isolates. As shown in Figure 4, the strains were separated based on the presence of the IncFIB-FIC(FII) plasmid-associated genes and then, within these groups, based on whether they carried *int, xis,* and *sopE* genes. In the 1217 strains that carry at least one of these genes, 1204 (98.9%) carry all three of the genes. The *int* and *xis* genes encode integrase and excisionase functions, respectively, which are associated with the integration and excision of bacteriophages from the bacterial chromosome [29]. The *int* and *xis* genes are also part of *Salmonella* Genomic Island 1 (SGI-1) which typically carries AMR and genes predicted to contribute to increased virulence [33,34]. Among the analyzed *S.* Schwarzengrund strains, none carried the SGE-1-associated genes (S004, S013–S026), while 61.3% and 61.1% carried *int* and *xis*, respectively. The highest percent of isolates carrying these two genes were those from chickens, with over 77% carriage (Table 2). Approximately a third of the isolates from human patients carried these genes. The presence of a high number of *int*/*xis* elements in the isolates from chickens and the lack of other SGE-1 genes may be evidence for the integration of chicken-associated phages into these strains [35]. The presence of phage genes was evident when examining the WGS assemblies of some of the strains, in that the genes flanking *int*/*xis* included those associated with phages (e.g., FSIS1609433, GenBank accession CP119491.1; FSIS1608447, accession CP119495.1). *sopE* also has a phage origin and encodes the guanine nucleotide exchange factor SopE, an effector for the SPI-1 type 3 secretion system (T3SS) [36,37]. SopE is translocated into the host cell via the T3SS where it interacts with the cytoskeleton to facilitate entry of the bacterium into the host [37]. In the two example strains note above, all three genes are located next to each other in their chromosomes. These features highlight the need for further examination of the role of integrated phages within the *S.* Schwarzengrund genome, as the strains isolated from blood and urine had an apparent higher prevalence of these phage-associated genes compared to those isolated from stool. Further research is needed to understand the role of integrated phages and the corresponding mobility of virulence genes in clinical and agricultural contexts.

As noted above, the IncFIB-FIC(FII) fusion plasmids carrying the aerobactin, and likely Sit, iron acquisition operons were common among isolates from chicken-related sources. It is important to note that all of the strains carried a chromosomally encoded Sit operon, which made it somewhat challenging to tease out when they are also located on plasmids. Related plasmids encoding iron acquisition operons (aerobactin and Sit) are common among avian pathogenic *Escherichia coli* (APEC), where they likely play a role in extraintestinal infections, and in *S.* Kentucky, where they may enhance the ability to colonize chickens [30,38]. These iron acquisition genes are important to overcome the innate immune response to APEC and *Salmonella* infections, which involves sequestration of iron from the pathogens [39,40]. Among the isolates from human patients in the current study, only about 10% carried one of the plasmids. The distribution of the plasmids is highlighted by Figure 3B, with the plasmid-containing group being predominately from chickens, while the groups lacking the plasmids had much more diverse sources, including most of those from human patients. The lack of diversity in the plasmid-carrying isolates was also borne out with the SNP typing, where most isolates carrying the fusion plasmids were of SNP type PDS000002218.839, two were of type PDS000112154.2, and four lacked SNP-type information (Figure S2C). Conversely, the strains lacking the plasmids represented a wide range of SNP types, the most common being PDS000027514.392; these plasmid-lacking strains were isolated from a number of different source types (including from blood, urine, and stool of human patients), states, and years of isolation (Table S1).

Among the strains collected from human patients, diverse virulence gene profiles were observed, suggesting that multiple factors may contribute to increased virulence in *S.* Schwarzengrund. For example, in the strains that were of SNP type PDS000002218.839 and carried the IncFIB-FIC(FII) plasmid, the aerobactin genes along with phage-associated genes were detected. A relatively high proportion (>20%) of these strains were isolated from urine, which may be related to the carriage of iron acquisition genes on the plasmids, which are also common among *E. coli* that cause urinary tract infections [40]. Among the fourteen isolates identified from blood (over half of all isolates from human patients lacked specimen types), 12 (86%) carried the *int, xis,* and *sopE* genes, compared to 53% (N = 50/96) of isolates from stool. There was also diversity among the fimbrial gene operons among many strains [41], with a group of SNP type PDS000001366.21 isolates from the U.K. carrying genes for the *stc, stf, stg,* and *stk* operons, while lacking the *sta* and *sti* operons carried by almost all (~98%) of the other strains.

It is important to note that this study is not without potential limitations, including the fact that it was reliant upon the data that is present in the GenBank database, which is reliant upon submissions from different investigators. This dependence could introduce bias, as submissions might be weighted towards strains of particular interest to the submitting investigator. Another challenge was the quality of the metadata, especially for isolates from human patients. In many cases, the type of sample (stool, blood, urine, etc.) or detailed geography (e.g., state) was not included in the available metadata, which limited the ability for more detailed examinations of virulence and clinical disease. Improving the quality of metadata in public databases is crucial for enabling more accurate epidemiological studies.

## 4. Conclusions

Overall, the results of this study provided a broad picture of the genetic diversity, especially as it relates to virulence factors, AMR genes, and plasmids, through the detailed analyses of WGS data from *S.* Schwarzengrund and lays the foundation for more extensive examination of the serotype. The serotype’s widespread presence in both human and food animal populations, combined with its ability to carry transmissible plasmids, poses significant public health challenges. Furthermore, the overlap in virulence profiles between human and food animal isolates highlights the potential zoonotic risk posed by *S.* Schwarzengrund and underscores its significance as a health concern. These findings emphasize the need for improved surveillance, particularly in the poultry industry, to mitigate the risk of AMR and virulence gene spread. Additionally, this study highlights the importance of enhancing metadata quality in public databases to facilitate more robust epidemiological investigations. Future studies should focus on characterizing the role of integrated phages and the potential for horizontal gene transfer in both clinical and agricultural settings.

## Figures and Tables

**Table 1:** Isolate demographics, including the source, country of origin, and year of isolation.

Isolate Source	Number	Percent	Country Isolated	Number	Percent	Year Isolated	Number	Percent
**Animal**	8	0.4	**Brazil**	23	1.1	**2022**	282	13.7
**Animal feed**	17	0.8	**Canada**	27	1.3	**2021**	217	10.5
**Avian**	13	0.6	**France**	4	0.2	**2020**	194	9.4
**Bovine**	62	3.0	**Germany**	5	0.2	**2019**	162	7.9
**Canine**	6	0.3	**India**	6	0.3	**2018**	325	15.8
**Chicken**	1145	55.6	**Taiwan**	32	1.6	**2017**	297	14.4
**Environmental**	21	1.0	**Thailand**	6	0.3	**2016**	151	7.3
**Fish**	10	0.5	**United Kingdom**	174	8.5	**2015**	86	4.2
**Food**	23	1.1	**United States**	1762	85.6	**2014**	78	3.8
**Human**	313	15.2	**Vietnam**	4	0.2	**2013**	32	1.6
**Porcine**	132	6.4	**Other**	9	0.4	**2012**	36	1.7
**Turkey**	300	14.6	**Not Reported**	6	0.3	**2011**	21	1.0
**Not Reported**	8	0.4	**Total:**	**2058**	**100**	**2001–2010**	157	7.6
**Total:**	**2058**	**100**				**<2000**	6	0.3
						**Not Reported**	14	0.7
						**Total**	**2058**	**100**

**Table 2:** Percentage of *S*. Schwarzengrund strains with the respective virulence gene by source type.

Source	Animal	Animal feed	Avian	Bovine	Canine	Chicken	Environmental	Fish	Food	Human	Porcine	Turkey	Not Reported
*allD*	12.5	5.9	0	1.6	0	0.8	14.3	20.0	13.0	8.3	0	0.3	0
*cdtB*	87.5	94.1	100	98.4	100	99.0	85.7	80.0	87.0	92.0	100	99.7	100
*envF*	12.5	5.9	0	1.6	0	0.8	14.3	20.0	13.0	8.6	0	0.3	0
*hlyE*	87.5	88.2	100	98.4	100	99.2	85.7	80.0	87.0	92.0	100	99.7	100
*icmF*	100	100	100	100	100	99.6	95.2	90.0	73.9	96.2	100	99.7	100
*int*	37.5	41.2	30.8	17.7	50.0	77.5	66.7	0	17.4	33.9	48.5	52.0	25.0
*iucA*	0	0	23.1	8.1	16.7	88.5	28.6	0	0	8.6	6.8	0.7	25.0
*iutA*	0	0	23.1	8.1	16.7	88.5	28.6	0	0	8.6	6.8	0.7	25.0
*lpfA*	12.5	0	0	0	0	0.8	14.3	20.0	13.0	1.9	0	0.3	0
*pagO*	100	100	100	100	100	99.8	90.5	90.0	87.0	93.6	100	100	100
*pltA*	87.5	94.1	100	98.4	100	99.0	85.7	80.0	87.0	92.0	100	99.7	100
*safA*	100	94.1	100	98.4	100	98.9	90.5	90.0	87.0	99.0	100	98.3	100
*sciL*	100	100	100	100	100	99.5	95.2	90.0	73.9	95.8	100	99.7	100
*sopE*	25.0	35.3	53.8	17.7	50.0	77.1	66.7	20.0	21.7	31.6	53.0	53.0	62.5
*sseK1*	0	0	0	0	0	0.3	9.5	10.0	13.0	8.0	0	0	0
*sspH2*	12.5	5.9	0	1.6	0	0.8	14.3	10.0	13.0	8.3	0.8	0.3	0
*staC*	87.5	94.1	100	98.4	100	99.2	85.7	80.0	87.0	92.0	100	99.7	100
*stbC*	100	100	100	100	100	97.4	100	100	100	100	100	100	100
*stcC*	12.5	0	0	0	0	0.6	14.3	20.0	13.0	7.7	0	0.3	0
*steC*	12.5	0	0	0	0	0.7	14.3	20.0	13.0	7.3	0	0.3	0
*stfC*	12.5	5.9	0	1.6	0	0.8	14.3	20.0	13.0	8.0	0	0.3	0
*stjC*	0	5.9	0	1.6	0	0.8	14.3	10.0	13.0	2.2	0	0	0
*stkC*	0	0	0	0	0	0.5	9.5	10.0	4.3	6.1	0	0	0
*tcfC*	100	94.1	100	98.4	100	99	95.2	100	82.6	88.5	100	100	100
*traT*	0	5.9	23.1	8.1	16.7	85.9	28.6	0	0	11.5	6.8	7.7	25.0
*xis*	37.5	41.2	30.8	17.7	50.0	77.4	66.7	0	17.4	33.2	48.5	52.0	0

Note: the color schema is based on the percentage of isolates with the respective gene, ranging from dark green (0%) to yellow (~25%) to orange (~50%) to dark red (100%).

## Data Availability

The data analyzed in this study is publicly available through GenBank and the corresponding accession numbers are identified in Table S1.

## Supplementary Materials

The following supporting information can be downloaded at https://www.mdpi.com/article/10.3390/microorganisms13010092/s1: Table S1: Sample Metadata for the strains examined in this study; Table S2: AMR Gene Analyses (1=present, 2=absent) provides the AMR gene profiles for all of the strains in this study; Table S3: Plasmid Transfer Gene Analyses (1=present, 2=absent) provides the plasmid transfer gene profiles for all of the strains in this study; Table S4: Virulence Gene Analyses (1=present, 2=absent) provides the virulence gene profiles for all of the strains in this study; Figure S1: Minimum spanning tree analyses based on the AMR and plasmid transfer gene profiles; Figure S2: Minimum spanning tree analyses based on the virulence genes of the *S.* Schwarzengrund strains included in this study. These trees are delineated based on isolation source, country of origin, and SNP cluster.
