# Supplementary material for: Salmonella enterica serovar Schwarzengrund: Distribution, Virulence, and Antimicrobial Resistance
Source: Microorganisms. 2025 Jan 6;13(1):92. doi: 10.3390/microorganisms13010092 (PMC11767468; doi:10.3390/microorganisms13010092)
Supplement: Supplementary file 1 [file microorganisms-13-00092-s001.zip › Schwarzengrund metaanalyses supplemental figures.pdf]

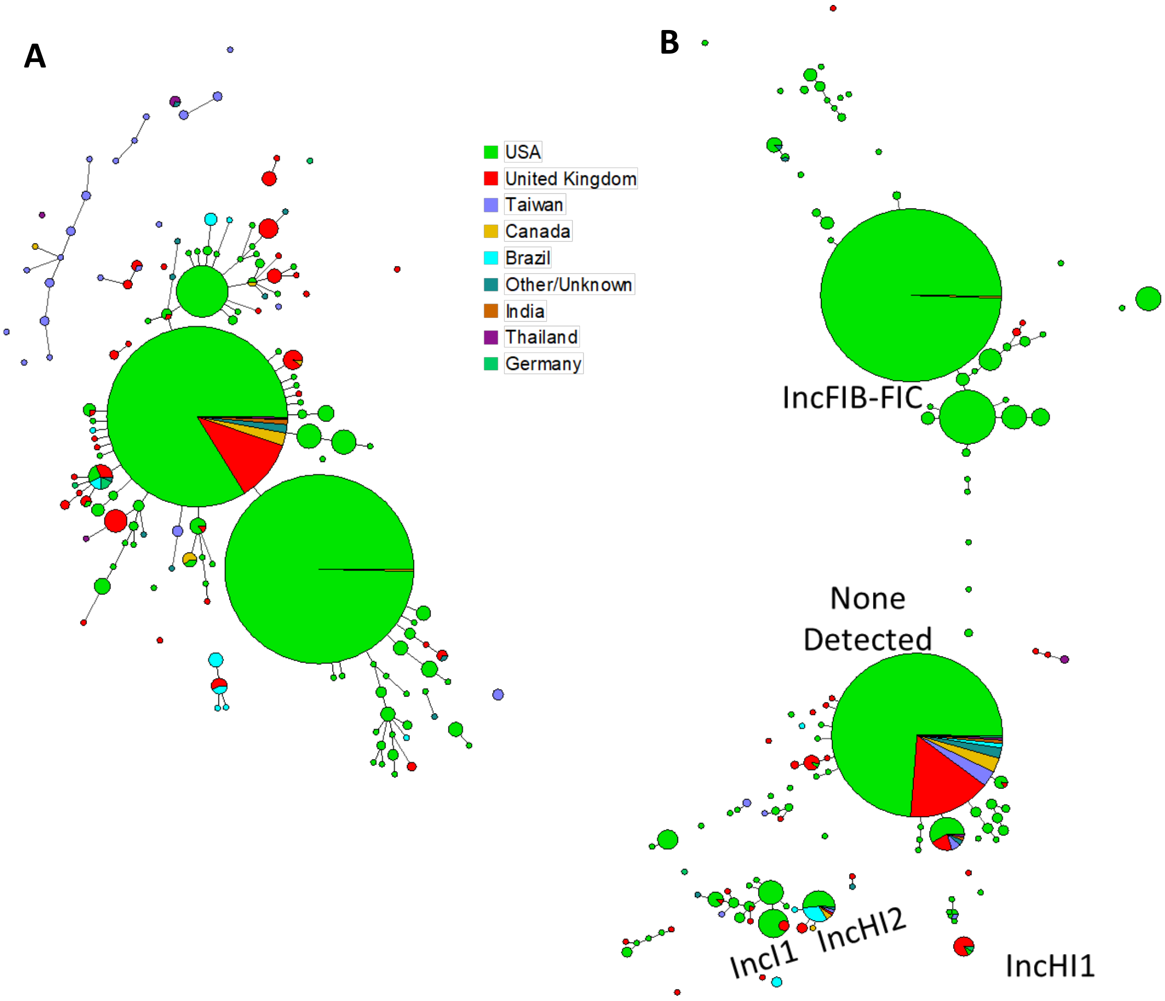

Figure S1. Minimum spanning tree analyses based on the AMR (panel A) and plasmid transfer gene (B) profiles of the *S.* Schwarzengrund strains included in the study. The trees are color coded based on country of origin. The relative size of the circles is proportional to the group size. In panel A, the larger circle in the middle represents the strains without an identified AMR gene. In panel B, the larger ball near the bottom is the group without a detected AMR plasmid. The groups representing the major plasmid types are annotated.

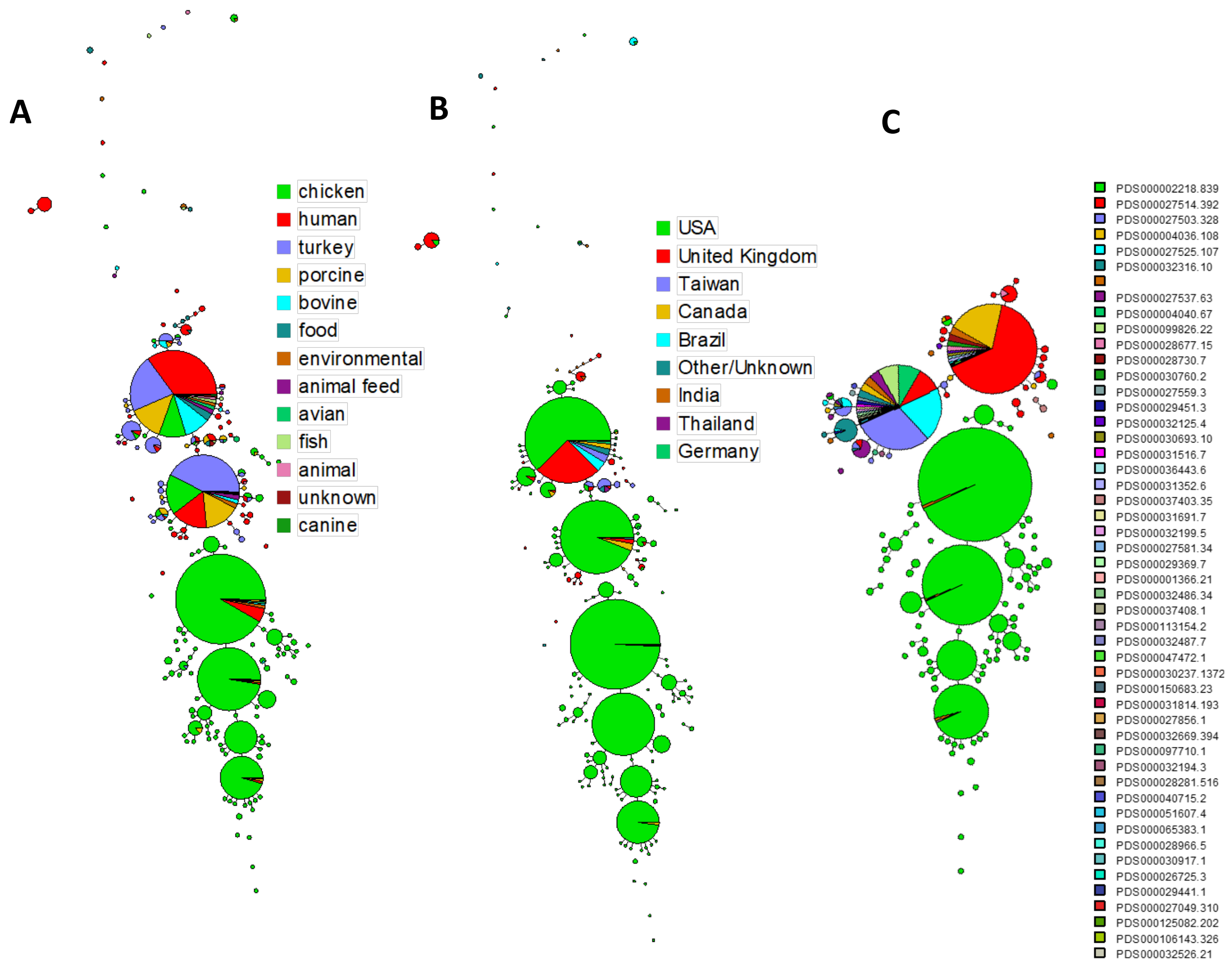

Figure S2. Minimum spanning tree analyses based on the phylogenetically relevant virulence genes of the *S.* Schwarzengrund strains included in the study. The trees are either color coded based on isolation source (panel A), country of origin (panel B) or SNP Cluster (panel C). The relative size of the circles is proportional to the group size.
